# Profiling of Redox-Active Lipophilic Constituents in Leaf Mustard (*Brassica juncea* (L.) Czern.) Cultivars Using LC-MS and GC-MS

**DOI:** 10.3390/antiox11122464

**Published:** 2022-12-14

**Authors:** Ramesh Kumar Saini, Ji-Woo Yu, Min-Ho Song, Hui-Yeon Ahn, Jung-Hoon Lee, Young-Soo Keum, Ji-Ho Lee

**Affiliations:** Department of Crop Science, Konkuk University, Seoul 143-701, Republic of Korea

**Keywords:** carotenoids, lutein, tocopherol, phytosterols, omega-3 fatty acids, phytochemicals, fatty acids, antioxidant activity

## Abstract

Leaf mustard is an important commercial and culinary vegetable. However, only limited information is available on the content and composition of the nutritionally important lipophilic constituents in these leaves. This research presents information on the contents and composition of carotenoids, tocols, phytosterols, and fatty acids in four cultivars of leaf mustard. The carotenoids and tocols were analyzed utilizing liquid chromatography (LC)–mass spectrometry (MS) with single ion monitoring (SIM), while phytosterols and fatty acids were analyzed using gas chromatography (GC)–MS and GC-flame ionization detection (FID), respectively. The LC-MS results revealed the dominance of (all-*E*)-lutein, within the range of 37.12 (*cv*. Asia Curled)—43.54% (*cv*. Jeok) of the total carotenoids. The highest amount of all of the individual carotenoids and total carotenoids (143.85 µg/g fresh weight; FW) were recorded in *cv*. Cheong. Among the studied leaf samples, 67.16 (*cv*. Asia Curled)—83.42 µg/g FW (*cv*. Cheong) of α-tocopherol was recorded. Among the phytosterols, β-sitosterol was the most dominant one among the studied mustard leaves, accounting for 80.42 (*cv*. Jeok)—83.14% (*cv*. Red frill) of the total phytosterols. The fatty acid analysis revealed the presence of a significant amount of rare hexadecatrienoic acid (C16:3n3) in the studied mustard leaves, which accounted for 27.17 (*cv*. Asia Curled)—32.59% (*cv*. Red frill) of the total fatty acids. Overall, the *cv*. Cheong represented the highest contents of carotenoids, tocols, and phytosterols. Moreover, *cv*. Red frill contains the highest amount of n-3 PUFAs and antioxidant compounds. Thus, these cultivars can be promoted in cuisines which can be eaten to obtain the highest health benefits.

## 1. Introduction

The epidemiological and clinical studies have shown the inverse relationship between the dietary consumption of fruits and vegetables and the prevalence of chronic diseases such as cancer, cardiovascular diseases (CVD), neurodegenerative diseases, obesity, type 2 diabetes (T2D), as well as all-cause mortality [1,2,3]. The bioactive antioxidant compounds, including the carotenoids and tocols present in fruits and vegetables, neutralize the cellular system’s free radicals, thus preventing the oxidative damage of cells, proteins, and DNA, resulting in a low incidence of related diseases [4,5,6].

Brassica vegetables are a rich source of carotenoids [7], tocopherols [8], and several other bioactive compounds [9]. *Brassica juncea* (L.) Czern., which is commonly known as leaf mustard, brown mustard, Korean leaf mustard, Oriental mustard, and vegetable mustard, is an annual herb belonging to the Brassicaceae family. The leaf mustard is widely used in Asian, African, Southern, and American cuisines.

Carotenoids are tetraterpenoid pigments that are universally synthesized by all photoautotrophs. In animals, provitamin A carotenoids (e.g., α- and β-carotene and β-cryptoxanthin) play essential roles by providing a dietary source of provitamin A. Additionally, in animals, the antioxidant activities of provitamin A and non-provitamin A carotenoids downregulate the pro-inflammatory mediators and oxidative stress and upregulate the cytoprotective phase II enzymes, thus protecting the consumer from cancer, neurodegenerative diseases, metabolic syndromes (T2D and CVD), and photooxidative damage to the eyes and skin [6,10].

Tocopherols and tocotrienols (α-, β-, γ-, and δ), which are collectively known as tocols, tocochromanols, or vitamin E) are vital components of cellular lipids, scavenging the free radicals, thus protecting the lipids from oxidative damage. With their crucial role in minimizing oxidative stress, a tocols-rich diet helps to minimize the incidence of CVD, neurodegenerative diseases, and several types of cancer [11,12,13].

Phytosterols (plant sterols) are another class of health-beneficial compounds that play a vital role in maintaining the blood level of low-density lipoprotein (LDL) cholesterol and endothelial function [14]. According to the U.S. Food and Drug Administration (FDA), the dietary intake of 2 g/d of non-esterified plant sterols is necessary to achieve the health benefits of lowering the blood total and LDL and reducing the risk of coronary heart disease (CHD) [15].

Leafy vegetables are not a significant source of fatty acids as they contain a small amount of total lipids (2–4%, dry weight) [16,17]. However, >50% of the total fatty acids in leafy vegetables are found in the form of α-linolenic acid (ALA, C18:3 cis-9,12,15), a type of polyunsaturated fatty acid (PUFA). In the body, the administrated ALA is used in the bioconversion to very long chain (VLC)-PUFAs that are beneficial to health, such as eicosapentaenoic (EPA) and docosahexaenoic acids (DHA) [18].

Most of the previous studies of the bioactive composition of leaf mustard have been focused on polyphenolic compounds [9,19], glucosinolates [20], and volatile components [21], while only a few studies are available on the composition of lipophilic bioactive compounds, such as carotenoids, tocopherols, phytosterols, and fatty acids. Moreover, the fatty acids and sterols were mainly investigated from seeds [22]. Furthermore, the bioactive composition may vary with the genetic, geographical, and environmental factors [23,24].

Considering the above facts, the present study aimed to investigate the composition of major lipophilic compounds, including carotenoids, tocols, sterols, and fatty acids in four cultivars of leaf mustard that are commonly consumed in Korea. The carotenoids and tocols were analyzed utilizing the liquid chromatography (LC)-single ion monitoring (SIM)-based mass spectrometry (MS) method. The phytosterols were analyzed by gas chromatography (GC)–MS, and the fatty acids were analyzed by GC-flame ionization detection (FID) and GC-MS. Moreover, the capabilities of scavenging free radicals were determined using DPPH^•^ and ABTS^•+^ assays. The carotenoids, tocols, phytosterols, and fatty acid composition data reported herein may help explore the bioactive potential of leaf mustard. Moreover, the knowledge of carotenoids, tocopherols, phytosterols, and fatty acids composition in different cultivars will be helpful in the selection of nutrient-dense cultivars for culinary preparations.

## 2. Materials and Methods

### 2.1. Plant Material, Reagents, and Standards 

The seeds of four *Brassica juncea* (L.) Czern. cultivars, Jeok (M1), Cheong (M2), Red frill (M3), and Asia Curled (M4) were obtained from Asia Seeds Co., Ltd., Seoul, Republic of Korea. The seeds were grown in the greenhouse at the ambient temperature and humidity (mean temperature of 10–15 °C) in March 2022–May 2022. Each cultivar was grown in three pots of 28 × 48 cm that were filled with a commercial potting mixture (Asia Seeds Co., Ltd., Seoul, Republic of Korea) containing 300–500 ppm NPK, and with a cation exchange capacity (CEC) of 30–100 cmol/kg.

**Figure 1:**
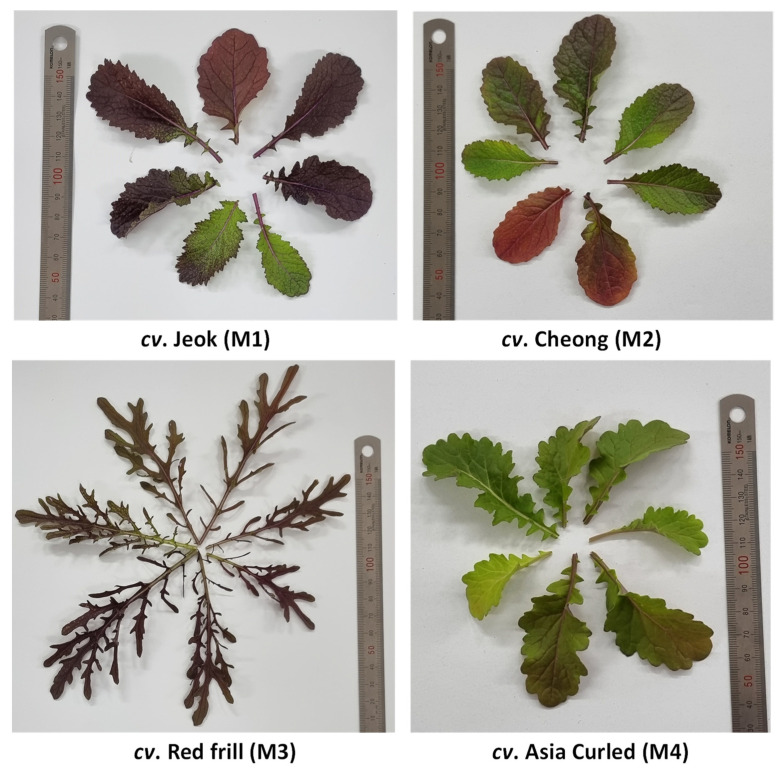
The phenotypic diversity among mustard cultivars used in this investigation.

After germination (a week after sowing), 18–20 plants were maintained in each pot. The plants were raised without applying any fertilizers and pesticides. Seven weeks after the showing, the healthy leaves from the entire plant were harvested, cleaned for the presence of foreign particles, and stored in a −80 °C deep freezer (Ilshin Biobase Co., Ltd., Dongducheonsi, Gyeonggido, Republic of Korea) until their analysis. Figure 1 shows the phenotypic diversity among the mustard cultivars used in this investigation.

Authentic standards of (all-*E*)-β-carotene, fatty acid standard mix (CRM47885), 5-β-cholestan-3α-ol (internal standard), β-sitosterol (24α-ethyl cholesterol), campesterol (24α-methyl cholesterol), 6-hydroxy-2,5,7,8-tetramethylchroman-2-carboxylic acid (Trolox), 2,2-diphenyl-1-picrylhydrazyl (DPPH^•^) and 2,2′-azino-bis(3-ethylbenzothiazoline-6-sulfonic acid) (ABTS^•+^) were obtained from Merck Ltd., Seoul, Republic of Korea. The tocols mix solution containing δ-, γ-, β-, and α-tocotrienol and δ-, γ-, β-, and α-tocopherol was purchased from ChromaDex, Inc., Irvine, CA, USA. The (all-*E*)-violaxanthin, 9-*Z*-neoxanthin, and (all-*E*)-lutein used in this study were purified from lettuce, and the (all-E)-zeaxanthin was purified from corn seeds using our established protocol [25].

All of the organic solvents used for extraction were of LC grade, and they were obtained from J.T. Baker^®^ (Avantor Performance Materials Korea Ltd.), Suwon-Si, Republic of Korea. 

### 2.2. Extraction of Crude Lipids (Lipophilic Compounds)

**Figure A1:**
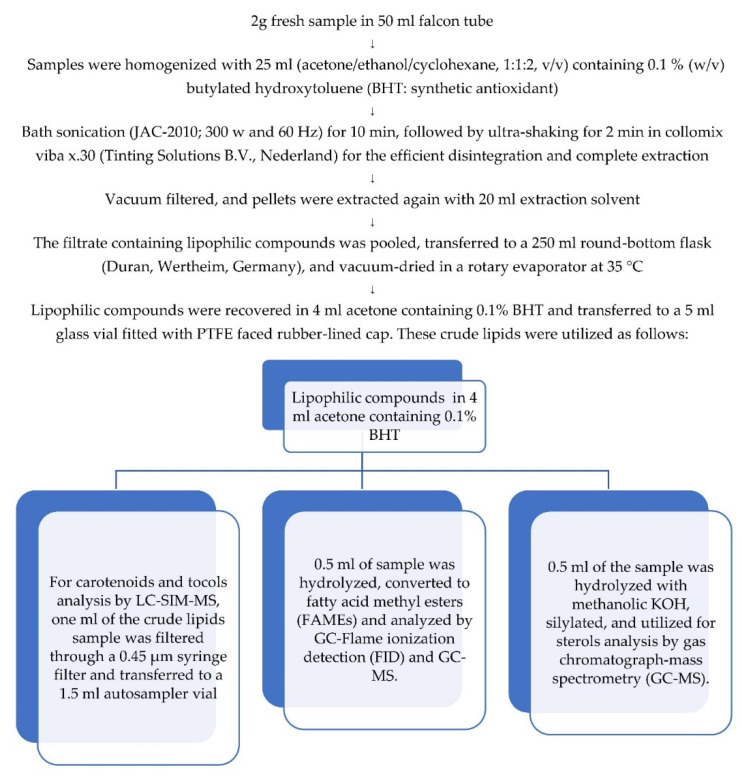
The outline of the method used for the extraction of lipophilic compounds from the mustard leaves.

The lipophilic compounds, including the carotenoids, tocols, sterols, and fatty acids, were extracted simultaneously from fresh mustard leaves following our optimized protocol [17] with minor modifications. The detailed extraction procedure is given in Appendix A Figure A1. Synthetic antioxidant butylated hydroxytoluene (BHT) was added to the extraction solvent (0.1% *w*/*v*) to prevent the degradation of the lipophilic compounds [26].

The tocols and carotenoids were simultaneously analyzed by liquid chromatography (LC)-selected-ion monitoring (SIM)-based tandem mass spectrometry (MS/MS) methods without hydrolysis as it can degrade these compounds [27].

**Figure A2:**
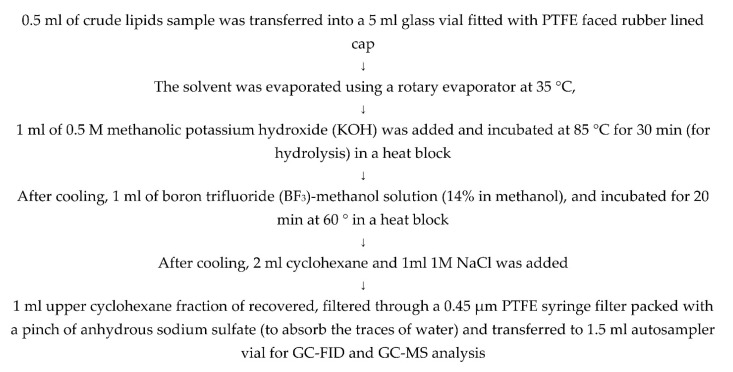
Methods used for the hydrolysis and the preparation of fatty acid methyl esters.

**Figure A3:**
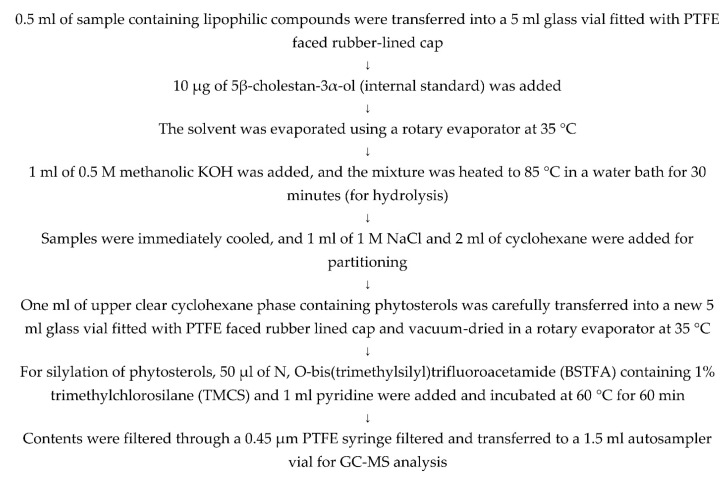
Method used for the hydrolysis and silylation of sterols for GC-MS analysis.

An aliquot of the extracted sample was hydrolyzed and converted to fatty acid methyl esters (FAMEs) (Figure A2) and trimethylsiloxy [−O-Si(CH3)3; TMS] derivatives (Figure A3) [27], and these were utilized for the GC-FID and GC-MS analyses of the fatty acids and phytosterols, respectively.

### 2.3. LC-SIM-MS Analysis of Tocols and Carotenoids

The major redox-active lipophilic constituents were analyzed by LC-SIM-based mass spectrometry method utilizing LCMS-9030 quadrupole time-of-flight (Q-TOF) mass spectrometer (Shimadzu, Tokyo, Japan). The LC-MS parameters used for the analysis of carotenoids and tocols are shown in Table 1. Additionally, the selected ion monitoring (SIM) parameters used for the analysis of carotenoids and tocols are shown in Table 2. 

### 2.4. Fames Determinations by GC-FID and GC-MS

The FAMEs were qualitatively analyzed by GC (Agilent 7890B, Agilent Technologies Canada, Inc., Mississauga, ON, Canada). The analytical conditions are shown in Table 3. For the accurate identification of the FAMEs, the mass spectrum was recorded using the QP2010 SE GS-MS (Shimadzu, Japan) following the GC-FID thermal program. The mass fragmentation pattern was compared with the authentic standards as well as reference databases (NIST08, NIST08S, and Wiley9) to confirm the identity of the FAMEs.

### 2.5. GC-MS Analysis of Sterols

The sterols were analyzed after silylation utilizing QP2010 SE GC-MS (Shimadzu, Tokyo, Japan). The analytical conditions are shown in Table 4. The identity of the sterols was confirmed by comparing their mass fragmentation patterns with the authentic standards as well as reference databases (Wiley9, NIST08, and NIST08S).

### 2.6. Antioxidant Activity

The antioxidant activities were analyzed using two different types of extracts. Extract one (containing lipophilic compounds) was prepared (without adding the BHT), as mentioned in Section 2.2. The second extract was prepared using aqueous methanol. To prepare this extract, two grams of fresh leaves of each cultivar were extracted with 25 mL of aqueous methanol (80% *v*/*v*) as described previously [17]. The ABTS^•+^ and DPPH^•^ radical scavenging abilities were determined according to our optimized method [28], based on Thaipong et al. [29]. The results were expressed in mg Trolox equivalents (TE)/100 g fresh weight (FW).

### 2.7. Statistical Analysis and Quality Control 

A total of four replicate extractions and analyses were performed for each leaf sample. The one-way analysis of variance (ANOVA) was performed using IBM SPSS statistics (version 25), taking into account a significance level of 0.05 (Tukey HSD).

The limits of detection (LOD) and limits of quantitation (LOQ) were determined according to the signal-to-noise (S/N) ratio between >3 and >10, respectively, for the quantitative analysis utilizing LC-MS [30].

The GC-MS method used for phytosterols analysis was recently validated [31]. The recoveries of the phytosterols were accurately monitored and normalized using 5β-cholestan-3α-ol as the internal standard (IS). 

## 3. Results and Discussion

### 3.1. Carotenoids and Tocols Composition

The solvent mixture with acetone/ethanol/hexane is most frequently employed to extract the polar and nonpolar carotenoids simultaneously [30]. In the preliminary investigations, we found that this solvent combination is effective for the simultaneous extraction of carotenoids, tocols, phytosterols, and fatty acids from mustard leaves. Moreover, considering the health and environmental hazards associated with hexane, this solvent was replaced with cyclohexane [32], which is safer and provided a similar yield of these lipophilic compounds from the studied samples (data not shown).

**Figure 2:**
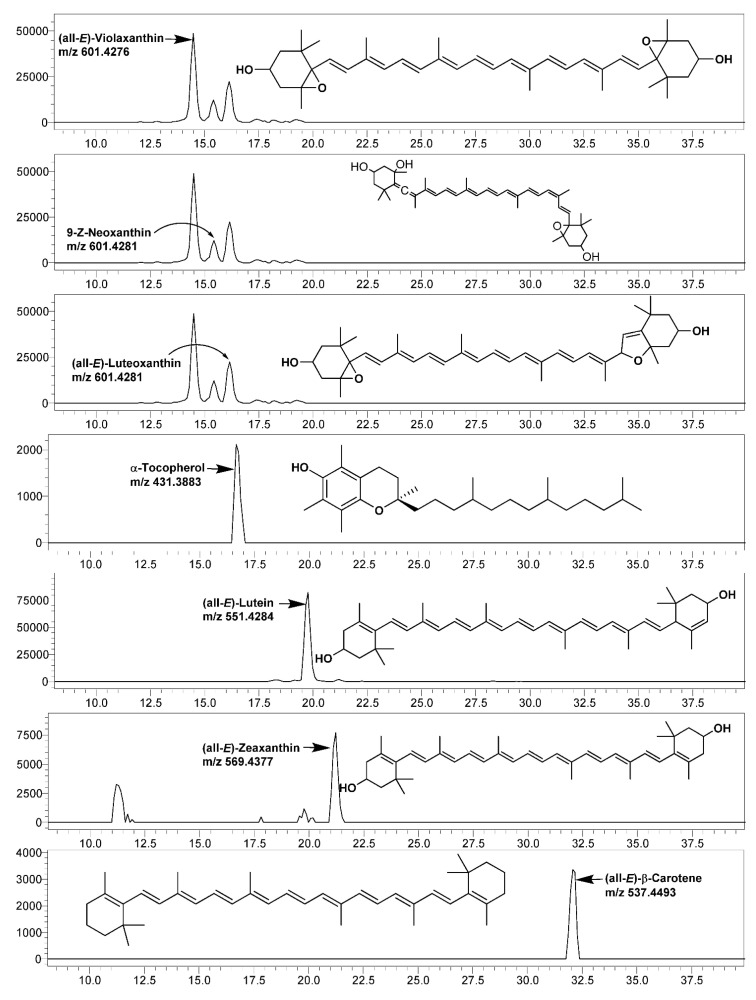
LC-single ion monitoring (SIM)-mass spectrometry (MS) chromatograms of carotenoids and α-tocopherol identified and quantified in leaves of various mustard cultivars.

In the present study, six major carotenoids, viz., (all-*E*)-violaxanthin, 9-Z-neoxanthin (all-*E*)-luteoxanthin, (all-*E*)-lutein, (all-*E*)-zeaxanthin, and (all-E)-β-carotene, were identified and quantified using the LC-SIM-based MS/MS method (Figure 2, Table 5). The obtained concentrations of all of the carotenoids were well above the LOQ (Table A1). 

Among the studied mustard leaves of four cultivars, (all-*E*)-lutein was the most dominating carotenoid, ranging between 37.12 (*cv*. Asia Curled) and 43.54% (*cv*. Jeok) of the total carotenoids, which was followed by (all-*E*)-β-carotene and (all-*E*)-violaxanthin. The highest amount of all of the individual carotenoids and total carotenoids (143.85 µg/g fresh weight; FW) were recorded in *cv*. Cheong.

In the present investigation, among all of the studied cultivars, the lutein contents were 2.1–2.6 times higher than the β-carotene ones were. However, previous studies on *B. juncea* leaves recorded significantly varied β-carotene and lutein levels. For instance, Frazie et al. [33] analyzed the carotenoid profile of leaves of eight *B. juncea* cultivars that were grown, including the Jeok cultivar analyzed in the present investigation. In this study, the β-carotene contents were 1.14 times higher than the lutein ones were in the mature leaves of the Jeok cultivar. Similarly, Farnham et al. [7] recorded 88.0–129.2 µg/g FW of β-carotene and 61.2–88.6 µg/g FW of lutein in the field-grown mature leaves of the *B. juncea* cultivars. In contrast, Zeb [34] recorded 124 µg/g FW of β-carotene and 184 µg/g FW of lutein in the *B. juncea* leaves marketed in Pakistan. 

We previously analyzed the carotenoid profile of 23 diverse lettuce cultivars and recorded 54.4–129.8 µg/g FW of the total carotenoids. Moreover, in that study, the (all-*E*)-β-carotene content ranged between 4.24 and 12.9 µg/g FW (7.30–13.03% of the total carotenoids), while in the present study, the (all-*E*)-β-carotene contents ranged between 21.34 and 24.45 µg/g FW (16.00—17.98% of the total carotenoids). This shows that mustard leaves are a richer source of provitamin A carotenoid (β-carotene) and total carotenoids than lettuce is.

The tocols include four naturally occurring tocopherols (δ-, γ-, β-, and α-) and four tocotrienols (δ-, γ-, β-, and α-) [13]. This study screened the mustard leaves for their tocols composition using LC-SIM-MS. In all of the studied samples, 67.16 (*cv*. Asia Curled)—83.42 µg/g FW (*cv*. Cheong) of α-tocopherol was recorded (Table 5), while other forms were not detected in a substantial amount. Only a few studies are available on the tocopherol contents of *B. juncea* leaves as most of the studies were focused on seed oil. Zeb [34] recorded 13.5 µg/g FW of α-tocopherol in the *B. juncea* leaves marketed in Pakistan. In contrast, Xiao et al. [8] recorded 221 µg/g FW of α-tocopherol in the commercially grown *B. juncea* microgreens. 

Attributable to the presence of high contents of bioactive antioxidant compounds, such as carotenoids and tocopherols in mustard leaf, their intake can minimize the oxidative stress-related diseases, including CVD, T2D, neurodegenerative disorders, and various types of cancer [35,36,37].

### 3.2. Phytosterol Contents

**Figure A4:**
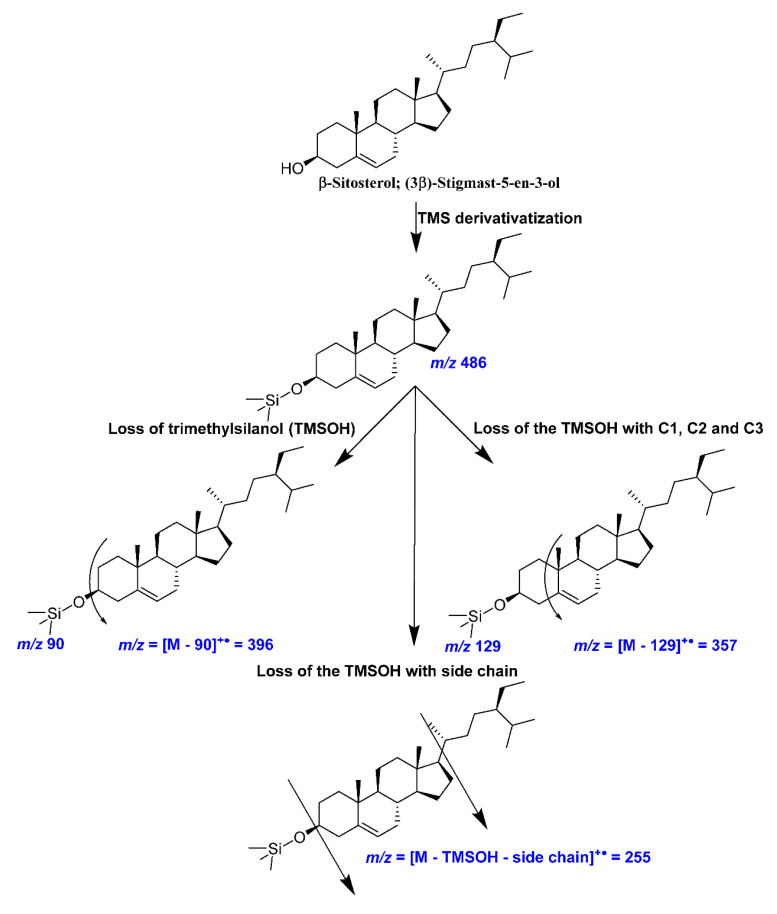
A representative mass fragmentation pattern of cholesterol (trimethylsiloxy (TMS) derivative) observed in the present investigation.

**Figure A5:**
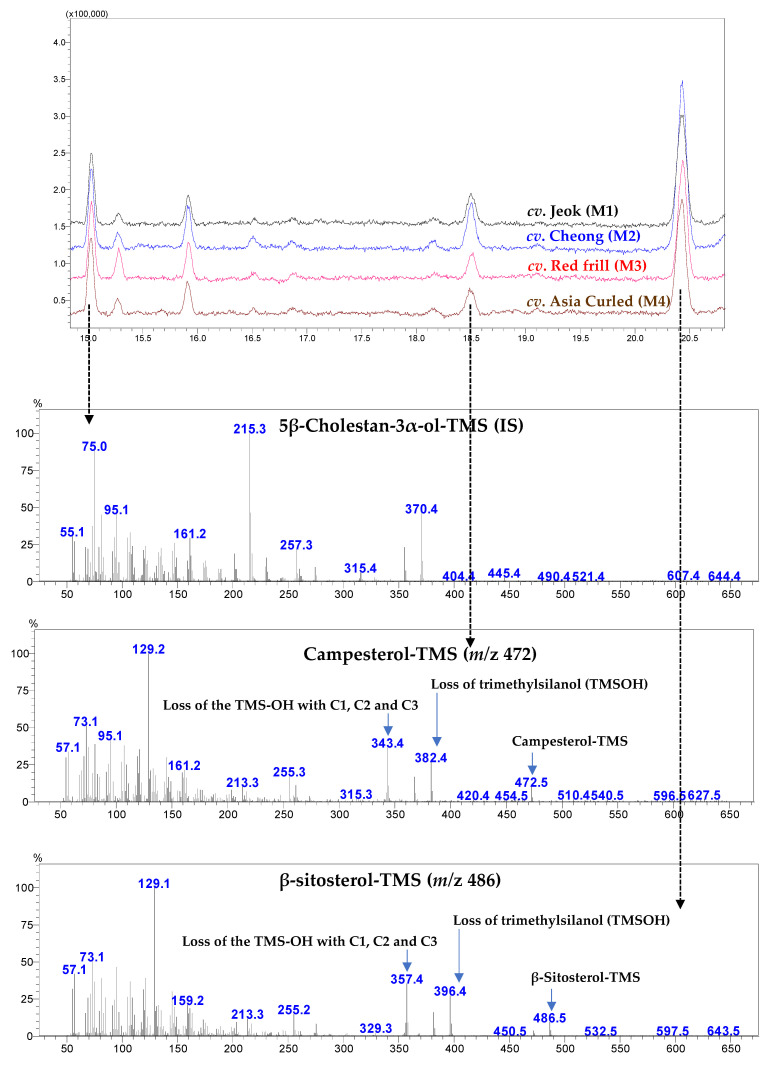
The representative gas chromatography (GC)–mass spectrometry (MS)-total ion chromatograms (TIC) of phytosterols identified in mustard leaves of various cultivars. IS: internal standard.

In this study, the phytosterols derivatized with trimethylsiloxy groups (−O-Si(CH_3_)_3_; TMS) and analyzed utilizing GC-MS revealed the presence of β-sitosterol (24α-ethyl cholesterol) and campesterol (24α-methyl cholesterol) in the mustard leaves (Figure A4 and Figure A5).

In the present study, β-sitosterol was the most dominant phytosterol among the studied mustard leaves, ranging between 151.39 (*cv*. Red frill) and 240.34 (*cv*. Cheong) µg/g FW, which accounted for 80.42 (*cv*. Jeok)—83.14% (*cv*. Red frill) of the total sterol (Table 6). Limited studies are available on the sterol composition of leaf mustard and other brassica family vegetables. Among the vegetables and fruits commonly consumed in China, β-sitosterol was the most dominating phytosterol, with the highest concentration of it (µg/g FW) in pea (414), cauliflower (408), and broccoli (345), while in rape (probably *Brassica napus*), 85 and 14 µg/g of β-sitosterol and campesterol was recorded, respectively [38].

We have previously recorded the dominance of β-sitosterol in herbs such as perilla (*Perilla frutescens* Britt.; 27.7–37.9 µg/g FW) [39] and *Kaempferia parviflora* Wall. Ex Baker (30.6–36.6 µg/g FW) [40] with campesterol as a minor phytosterol.

The results of the present investigation and previous report suggest that the Brassica family vegetables, including leaf mustard, are good sources of bioactive phytosterols.

### 3.3. Fatty Acids

Herbs (photosynthetic tissue) are not a significant source of fatty acids as they are generally deficient in total lipids (2–4%, dry weight) [18]. However, herbs contain high proportions of health-beneficial omega-3 (n-3) fatty acid in the form of α-linolenic (ALA; C18:3n3).

**Figure 3:**
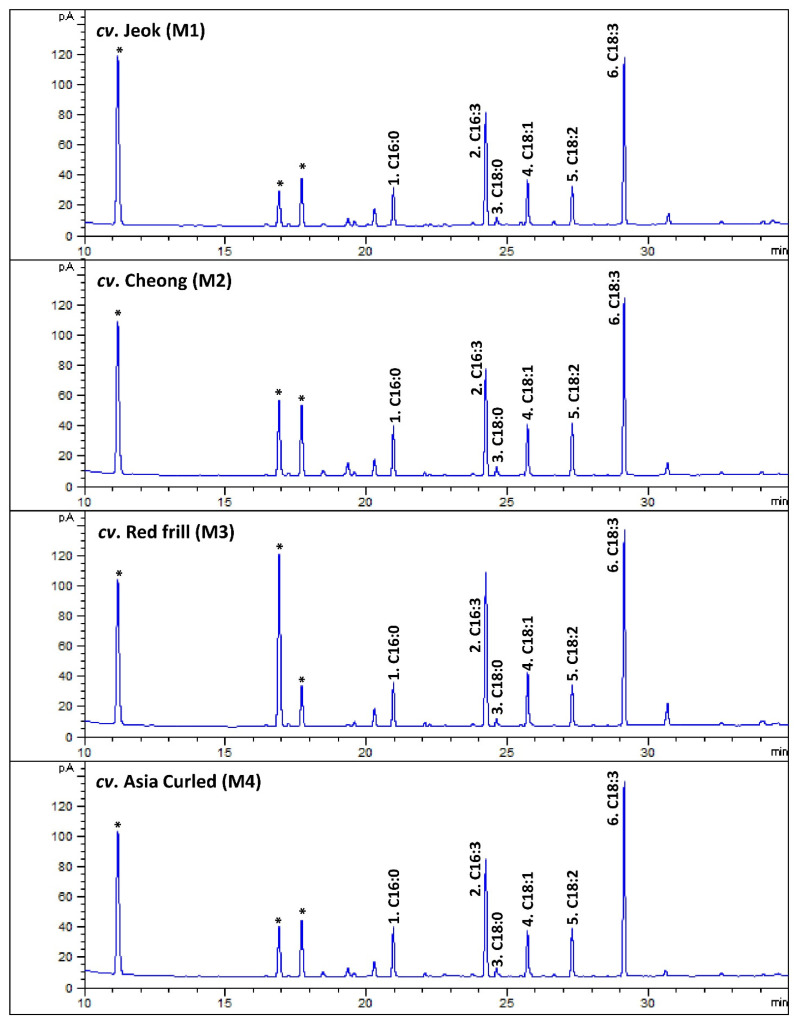
The representative gas chromatography (GC)-flame ionization detection (FID) chromatograms of fatty acids methyl esters (FAMEs) of leaves of various mustard cultivars. The peak numbers (from 1 to 6) are correspondent to Table 7. * Not a FAME.

In the present study, six major fatty acids were identified from the mustard leaf, and their relative occurrence (percentages of the total fatty acids) were estimated (Figure 3; Table 7). Among the studied mustard leaves, ALA was the most dominant fatty acid, accounting for 36.09 (*cv*. Cheong)—38.98% (*cv*. Asia Curled) of the total fatty acids. The previous reports also state that ALA is the most abundant fatty acid in herbs (e.g., green leafy vegetables), such as cabbage and spinach [41], perilla (*Perilla frutescens* Britt.) [39], lettuce [17], and Komatsuna (Japanese mustard spinach; *Brassica rapa var*. perviridis), and Tatsoi (*Brassica rapa var*. rosularis) [42].

**Figure A6:**
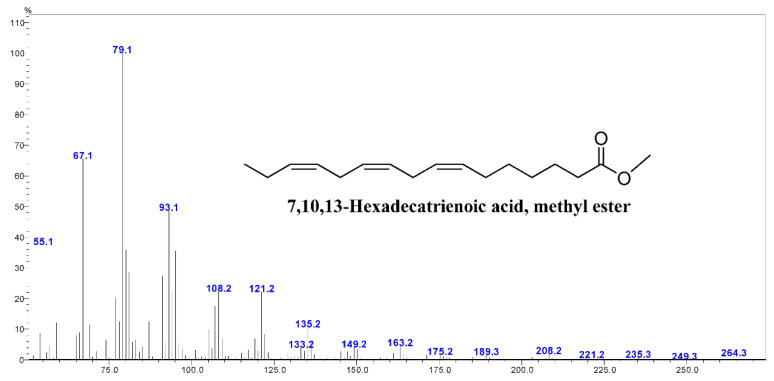
The GC-mass spectrum of 7,10,13-hexadecatrienoic acid identified in mustard leaves.

In contrast to other herbs, a significant amount of hexadecatrienoic acid (C16:3n3; all-cis 7,10,13) was recorded from the studied mustard leaves, accounting for 27.17 (*cv*. Asia Curled)—32.59% (*cv*. Red frill) of the total fatty acids. The GC–mass spectrum of the hexadecatrienoic acid identified from the mustard leaves is given in Figure A6.

The presence of a substantial amount of hexadecatrienoic acid is rare in higher plants. However, it has been previously recorded in the photosynthetic tissue of *Brassica napus* L. and *Brassica oleracea* L., which are classified as “16:3” plants (containing >2% of C16:3 fatty acids in total lipids), which is part of primitive lipid metabolism [43]. Interestingly, the contents of polyunsaturated fatty acids (PUFAs) in the photosynthetic leaves of *B. napus* L. are largely influenced by the environmental temperatures [24]. A low temperature induces fatty acid desaturation in *B. napus* L., resulting in an enhanced accumulation of C18:3 and C16:3 fatty acids, which helps maintain the fluidity in the membrane at a low temperature [24]. A substantially high amount of hexadecatrienoic acid recorded in the present investigation was probably a result of the low temperature (mean temperature of 10–15 °C) during the growth of the mustard plants.

From the nutritional perspective, the dominance of ALA and hexadecatrienoic acid in the mustard leaves makes them exceptionally rich in total PUFAs, containing 74.99–78.28% of the total fatty acids. Moreover, the total PUFAs/total saturated fatty acids (SFAs) were high (5.63 in *cv*. Cheong—7.26 in *cv*. Red frill). 

Omega-3 (n-3) PUFAs are essential for normal growth and development. Moreover, they have vital positive effects on the brain, eyes, heart, joints, skin, mood, and behavior [18]. Thus, consuming mustard leaves for major nutritionally vital phytoconstituents (e.g., vitamins) and high proportions of n-3 PUFAs (63.89–69.74% of total fatty acids) may be one of their health benefits. 

### 3.4. Antioxidant Activity

In the present study, the antioxidant activities were analyzed from two different types of extracts. Interestingly, the aqueous methanolic extract showed 2–5-fold more ABTS^•+^ and DPPH^•^ radical scavenging activities compared to the lipophilic extract prepared using acetone/ethanol/cyclohexane (1:1:2, *v*/*v*; recovered in acetone) (Table 8). The previous studies have also revealed that methanol/water extract provides better ABTS^•+^ and DPPH^•^ radical scavenging activities from herbs than the extracts do which were obtained with acetone [44,45]. In the investigations of the antioxidant activity of *Daphne gnidioides* L. and *Daphne sericea* L. leaves, the methanol extracts showed 2–3-fold more ABTS^•+^ and DPPH^•^ radical scavenging compared to the acetone extract. Similarly, from the *Amaranthus* leaf, the methanol/water extract showed significantly higher antioxidant activities and total phenolic contents (TPC) than the extract did that was prepared in acetone. 

In herbs, the phenolic compounds (flavonoids and phenolic acids), tocopherols, ascorbic acid, and carotenoids are major antioxidants [46]. The present study used an acetone/ethanol/cyclohexane-based solvent to extract the lipophilic compounds, including carotenoids, tocopherols, sterols, and fatty acids. However, the significantly highest radical scavenging activities obtained from the aqueous methanolic extract were probably the results of a higher extraction of polyphenolic compounds. 

The present study recorded the highest contents of carotenoids and α-tocopherol from *cv*. Cheong. However, the aqueous methanolic prepared from *cv*. Red frill showed significantly more ABTS^•+^ (640.66 mg TE/100 g FW) and DPPH^•^ radical scavenging (284.96 mg TE/100 g FW) activities (Table 8). In contrast, among the lipophilic extracts prepared using acetone/ethanol/cyclohexane, *cv*. Cheong showed significantly higher ABTS^•+^ activity values of 148.27 mg TE/100 g FW (Table 8). Interestingly, the α-tocopherol content (Table 5) was also highest in this cultivar (*cv*. Cheong). Moreover, the α-tocopherol contents showed a significant positive correlation (*R* = 0.796) with the ABTS^•+^ activity of the lipophilic extract (Table 9), which suggests that the α-tocopherol contents present in the mustard leaves are efficient scavenger of ABTS^•+^ radicals. In addition, the phytosterol contents correlate well (*R* = 0.949) with the α-tocopherol contents (Table 9). Surprisingly, in the present study, the total carotenoid contents were not correlated with the antioxidant activities (Table 9), suggesting that bioactives other than the carotenoids are more potent antioxidants in the mustard leaves.

Both the ABTS^•+^ and DPPH^•^ radical scavenging assays employed in the present study are based on the electron transfer (ET) mechanism. In agreement with previous reports [29,47], a significant correlation was obtained between these assays (Table 9). 

The Trolox equivalent antioxidant capacity (TEAC) in Brassica vegetables has shown a significant positive correlation with the anthocyanins contents [48]. Thus, the significantly highest radical scavenging activities obtained from *cv*. Red frill was probably the result of a high accumulation of anthocyanin pigments—the intense red color of *cv*. Red frill leaves, compared to other studied cultivars (Figure 1), also suggest a presence of a substantial amount of anthocyanins.

## 4. Conclusions

The results of the present study indicate that the contents of carotenoids, α-tocopherol, phytosterols, and fatty acids varied significantly among the cultivars. Among the mustard leaves of four cultivars studied, the highest contents of the total carotenoids (143.85 µg/g FW), α-tocopherol (83.42 µg/g FW), and total phytosterols (294.30 µg/g FW) were recorded in *cv*. Cheong.

Interestingly, the fatty acid analysis revealed the presence of a significant amount of rare hexadecatrienoic acid (C16:3n3) in the studied mustard leaves, accounting for 27.17 (*cv*. Asia Curled)—32.59% (*cv*. Red frill) of the total fatty acids. Moreover, the dominance of α-linolenic (ALA; C18:3n3) and hexadecatrienoic acid in the mustard leaves makes them exceptionally rich in total omega-3 (n-3) PUFAs, with the highest contents of 69.74% in *cv*. Red frill.

Overall, the *cv*. Cheong represented the highest contents of carotenoids, tocols, and phytosterols. Moreover, *cv*. Red frill contains the highest amount of n-3 PUFAs and antioxidant compounds. Thus, the use of these cultivars can be promoted in cuisines which can be eaten to obtain the highest health benefits. Further studies may be beneficial in revealing the hydrophilic constituents, including anthocyanin, in leaf mustard cultivars.

## Figures and Tables

**Table 1:** LC-MS parameters used for the analysis of carotenoids and tocols.

HPLC: Nexera 40 Series UHPLC (Shimadzu, Tokyo, Japan)	
Column	YMC C30 carotenoid column (150 mm × 4.6 mm, 3 μm; YMC, Wilmington, NC)
Column oven temperature	20 °C
Mobile phase	A—methanol/water (95/5; *v*/*v*) containing 5 mM ammonium formate; B—methyl tertiary butyl ether (MTBE)/methanol/water (90/7/3, *v*/*v*/*v*), containing 5 mM ammonium formate.
Flow rate	0.5 mL/min
Injection volume	2 µL
Gradient program	0% B (0 min)–100% B (45 min); 5 min post-run (0% B)
MS: LCMS-9030 quadrupole time-of-flight spectrometer (Shimadzu, Tokyo, Japan)	
Ionization	Atmospheric pressure chemical ionization (APCI; Positive)
Nebulizing gas flow	3 L/min
Interface temperature	400 °C
Drying gas flow	10 L/min
DL temperature	300 °C
Heat block Temperature	300 °C
Corona needle voltage	4.0 kv
MS program	0.0 min—diverter valve to drain; 8.0 min—diverter valve to MS.
Screening mode	Single ion monitoring (SIM)
Data acquisition (sampling)	1.85625 Hz
Q1 resolution	±20 ppm

**Table 2:** Selected ion monitoring (SIM) parameters used for the analysis of carotenoids and tocols.

Compound Class	Compound	Transition (*m/z*)
Tocols	δ-tocotrienol	397.3113
	γ-tocotrienol	411.3268
	δ-tocotrienol	411.3268
	α-tocotrienol	425.3423
	δ-tocopherol	402.3488
	γ-tocopherol	416.3669
	δ-tocopherol	416.3669
	α-tocopherol	431.3883
Carotenoids	(all-*E*)-violaxanthin	601.4276
	9-*Z*-neoxanthin	601.4281
	(all-*E*)-luteoxanthin	601.4281
	(all-*E*)-lutein	551.4284
	(all-*E*)-zeaxanthin	569.4377
	(all-*E*)-β-carotene	537.4493

**Table 3:** GC-FID parameters used for the analysis of fatty acids.

Parameters	Value		
Injection temperature	250 °C		
Injection mode	Split (5:1)		
Injection volume	1 µL		
Split flow	10 mL/min		
Inlet pressure	54.901 psi		
Inlet total flow	15 mL/min		
Septum purge flow	3 mL/min		
Carrier Gas Flow	2 mL/min (Nitrogen)		
Detector H_2_ flow	30 mL/min		
Detector airflow	400 mL/min		
Detector makeup flow	25 mL/min (Nitrogen)		
Column	SP-2560 capillary (100 m, 0.20 μm film thickness, 0.25 mm ID; Merck KGaA, Darmstadt, Germany).		
Column oven temperature	Rate (°C/min)	Final temperature (°C)	Hold time (min)
	-	140	1
	5	240	15
Detector	Flame ionization detector (FID)		
Detector temperature	260 °C		
Run time	45 min		
Post run time	5 min		

**Table 4:** GC-MS parameters used for the analysis of sterols.

Gas Chromatograph			
Column over temperature	150 °C		
Injection Temperature	260 °C		
Injection mode	split		
Carrier gas	Helium		
Flow control mode	Liner velocity		
Pressure	86.5 kPa		
Total Flow	8.6 mL/min		
Column Flow	0.93 mL/min		
Liner velocity	36.7 cm/s		
Purge flow	3.0 mL/min		
Column	DB-5 ms (30 m, 0. 25 μm film thickness, 0.25 mm ID; Agilent 7890B, Agilent Technologies Canada, Inc		
Column oven temperature	Rate (°C/min)	Final temperature (°C)	Hold time (min)
	-	150	1
	20	300	30
Total program time	38.5 min		
Mass spectrometer			
Ion source temperature	260 °C		
Interface temperature	280 °C		
Solvent cut time	3 min		
Start time	6 min		
End time	38 min		
Acquiring mode	Scan		
Event time	30 s		
Scan speed	2500		
Start *m*/*z*	50.00		
End *m*/*z*	650.00		

**Table 5:** The contents of carotenoids and α-tocopherol in leaves of various mustard cultivars.

Compounds	*cv*. Jeok (M1)	*cv*. Cheong (M2)	*cv*. Red Frill (M3)	*cv*. Asia Curled (M4)
(all-*E*)-violaxanthin	22.98 ± 2.98 ^a^	23.86 ± 2.45 ^a^	10.92 ± 0.92 ^b^	22.08 ± 1.99 ^a^
9-*Z*-neoxanthin	10.35 ± 0.03 ^b^	12.62 ± 0.92 ^a^	12.75 ± 0.75 ^a^	12.99 ± 0.07 ^a^
(all-*E*)-luteoxanthin	10.65 ± 0.65 ^c^	19.94 ± 1.94 ^a^	15.80 ± 1.80 ^b^	20.43 ± 2.34 ^a^
(all-*E*)-lutein	53.63 ± 0.38 ^b^	59.17 ± 3.22 ^a^	52.76 ± 2.76 ^b^	50.48 ± 0.85 ^b^
(all-*E*)-zeaxanthin	4.22 ± 0.14 ^c^	5.23 ± 0.44 ^b^	9.70 ± 0.31 ^a^	5.55 ± 0.62 ^b^
(all-*E*)-β-carotene	21.34 ± 0.26 ^b^	23.02 ± 0.18 ^ab^	21.90 ± 1.90 ^ab^	24.45 ± 0.89 ^a^
Total carotenoids	123.17 ± 2.80 ^b^	143.85 ± 4.25 ^a^	123.83 ± 7.82 ^b^	135.97 ± 0.16 ^a^
α-tocopherol	76.83 ± 5.33 ^ab^	83.42 ± 3.36 ^a^	68.68 ± 0.81 ^b^	67.16 ± 8.48 ^b^

Values (µg/g FW) are the mean ± standard deviation of four replicates. Different superscript letters (i.e., a, b, and c) indicate statistically significant differences among the different cultivars (*p* < 0.05, Tukey HSD).

**Table 6:** The phytosterol contents in leaves of various mustard cultivars.

Phytosterol	RT (min)	*cv*. Jeok (M1)	*cv*. Cheong (M2)	*cv*. Red Frill (M3)	*cv*. Asia Curled (M4)
Campesterol	18.509	49.13 ± 4.79 ^a^	53.96 ± 4.86 ^a^	30.70 ± 2.71 ^b^	36.06 ± 3.31 ^b^
β-Sitosterol	20.438	201.84 ± 19.2 ^b^	240.34 ± 3.76 ^a^	151.39 ± 14.1 ^d^	174.25 ± 5.16 ^c^
Total phytosterol		250.97 ± 23.8 ^b^	294.30 ± 8.52 ^a^	182.09 ± 16.4 ^c^	210.31 ± 8.21 ^c^

Values (µg/g FW) are mean ± standard deviation of four replicates. RT: retention time. Different superscript letters (i.e., a, b, and c) indicate statistically significant differences among the different cultivars (*p* < 0.05, Tukey HSD).

**Table 7:** Fatty acid composition of leaves of various mustard cultivars.

Peak No	FAME	RT (min)	*cv*. Jeok (M1)	*cv*. Cheong (M2)	*cv*. Red Frill (M3)	*cv*. Asia Curled (M4)
1	C16:0 (Palmitic)	20.992	9.01 ± 1.20 ^b^	11.09 ± 0.37 ^a^	9.18 ± 0.15 ^b^	10.82 ± 0.10 ^a^
2	C16:3n3 (Hexadecatrienoic acid)	24.270	32.04 ± 3.25 ^a^	27.80 ± 2.50 ^b^	32.59 ± 0.74 ^a^	27.17 ± 0.60 ^b^
3	C18:0 (Stearic)	24.652	2.13 ± 0.45 ^a^	2.25 ± 0.18 ^a^	1.56 ± 0.03 ^b^	2.28 ± 0.12 ^a^
4	C18:1n9c (Oleic)	25.746	10.57 ± 0.84 ^b^	11.67 ± 0.38 ^a^	11.33 ± 0.06 ^ab^	10.53 ± 0.25 ^b^
5	C18:2n6c (Linoleic)	27.334	8.56 ± 0.88 ^b^	11.10 ± 0.43 ^a^	8.19 ± 0.10 ^b^	10.22 ± 0.10 ^a^
6	C18:3n3 (α-Linolenic)	29.180	37.68 ± 0.28 ^b^	36.09 ± 1.23 ^c^	37.14 ± 0.43 ^bc^	38.98 ± 0.29 ^a^
	∑SFAs		11.15 ± 1.37 ^b^	13.34 ± 0.53 ^a^	10.74 ± 0.16 ^b^	13.10 ± 0.06 ^a^
	∑PUFAs		78.28 ± 2.19 ^a^	74.99 ± 0.88 ^b^	77.93 ± 0.21 ^a^	76.37 ± 0.25 ^ab^
	∑n-3 PUFAs		69.72 ± 3.06 ^a^	63.89 ± 1.29 ^b^	69.74 ± 0.31 ^a^	66.15 ± 0.31 ^b^
	∑PUFAs/∑SFAs		7.12 ± 1.07 ^a^	5.63 ± 0.29 ^b^	7.26 ± 0.13 ^a^	5.83 ± 0.03 ^b^

Values (mean ± standard deviation) are percentages of the total fatty acids from four determinations. FAME: fatty acid methyl ester; RT: retention time; PUFAs: total polyunsaturated fatty acids; SFAs: total saturated fatty acids. Different superscript letters (i.e., a, b, and c) indicate statistically significant differences among the different cultivars (*p* < 0.05, Tukey HSD).

**Table 8:** The antioxidant activity of leaves of various mustard cultivars.

Antioxidant Assay	*cv*. Jeok (M1)	*cv*. Cheong (M2)	*cv*. Red Frill (M3)	*cv*. Asia Curled (M4)
ABTS^•+^ activity ^1^	535.67 ± 21.28 ^c^	580.44 ± 22.35 ^b^	640.66 ± 19.14 ^a^	515.49 ± 6.62 ^c^
ABTS^•+^ activity ^2^	133.14 ± 4.72 ^b^	148.27 ± 3.31 ^a^	133.48 ± 4.11 ^b^	100.73 ± 3.15 ^c^
DPPH^•^ activity ^1^	192.89 ± 6.48 ^c^	243.14 ± 1.29 ^b^	284.96 ± 10.96 ^a^	170.20 ± 2.12 ^d^
DPPH^•^ activity ^2^	63.74 ± 4.74 ^bc^	74.95 ± 3.40 ^b^	80.76 ± 2.94 ^a^	60.23 ± 2.56 ^c^

Values (mg of Trolox equivalent/100 g FW) are mean ± standard deviation of four replicates. Different superscript letters (i.e., a, b, and c) indicate statistically significant differences among the different cultivars (*p* < 0.05, Tukey HSD). ^1^ Activity from 80% methanolic extract (*v*/*v*); ^2^ Antioxidant activity from lipophilic extract prepared using acetone/ethanol/cyclohexane (1:1:2, *v*/*v*).

**Table 9:** The correlation coefficient (*R*) between total carotenoids, α-tocopherol, and phytosterol contents and the antioxidant potential of leaves of various mustard cultivars.

	Total Carotenoids	α-Tocopherol	Total Phytosterol	ABTS^•+^ Activity ^1^	ABTS^•+^ Activity ^2^	DPPH^•^ Activity ^1^	DPPH^•^ Activity ^2^
Total carotenoids	1.000						
α-tocopherol	0.454	1.000					
Total phytosterol	0.609	0.949	1.000				
ABTS^•+^ activity ^1^	−0.226	−0.002	−0.286	1.000			
ABTS^•+^ activity ^2^	0.069	0.796	0.567	0.574	1.000		
DPPH^•^ activity ^1^	−0.141	0.116	−0.168	0.992	0.657	1.000	
DPPH^•^ activity ^2^	−0.050	0.180	−0.094	0.978	0.687	0.995	1.000

^1^ Antioxidant activity from 80% methanolic extract (*v*/*v*); ^2^ antioxidant activity from lipophilic extract prepared using acetone/ethanol/cyclohexane (1:1:2, *v*/*v*).

## Data Availability

Data is contained within the article.

## Appendix A

**Table A1:** Limits of detection (LOD; Lower limit of detection) and limits of quantitation (LOQ; Lower limit of quantitation) of carotenoids and tocols analyzed utilizing LC-MS/MS.

S/N	Compound Class	Compound	Ret. Time	LOQ (µg/g)	LOD (µg/g)
1	Carotenoids	(all-*E*)-violaxanthin	14.496	0.79	0.26
2		9-*Z*-neoxanthin	15.413	3.21	1.07
3		(all-*E*)-luteoxanthin	16.000	0.35	0.12
4		(all-*E*)-lutein	19.738	0.35	0.12
5		(all-*E*)-zeaxanthin	21.280	0.10	0.03
6		(all-*E*)-β-carotene	32.179	0.36	0.12
7	Tocols	α-tocopherol	16.705	1.34	0.44
